# Histological Evaluation of *Mentha spicata* Essential Oil in a Rat Excisional Wound Model with Network-Based Mechanistic Insights

**DOI:** 10.3390/biomedicines14040739

**Published:** 2026-03-24

**Authors:** Cafer Yildirim, Nihal Kayir, Merve Gulsen Bal Albayrak, Ayse Hande Yozgat, Durul Seyma Sen

**Affiliations:** 1 Department of Basic Medical Sciences, Faculty of Dentistry, Ankara University, Ankara 06560, Turkey; 2 Department of Medical Pharmacology, Faculty of Medicine, İstanbul Medipol University, İstanbul 34820, Turkey; nihalkayirr@gmail.com; 3 Department of Molecular Gastroenterology and Hepatology, Gastroenterology and Hepatology Institute, Kocaeli University, Kocaeli 41001, Turkey; merve.bal@kocaeli.edu.tr; 4 Department of Histology and Embryology, Faculty of Medicine, Ankara University, Ankara 06560, Turkey; hyozgat@ankara.edu.tr (A.H.Y.); sends@ankara.edu.tr (D.S.S.)

**Keywords:** *Mentha spicata*, wound healing, rat, essential oil, olive oil, inflammation, histology, GC–MS, network pharmacology, GPCR signaling

## Abstract

**Background/Objectives**: Wound healing is a complex biological process involving inflammatory, proliferative, and remodeling phases. Plant-derived essential oils are increasingly investigated as topical therapeutic agents, although their biological effects are strongly influenced by composition and formulation. The present study evaluated the effects of topical *Mentha spicata* essential oil on cutaneous wound healing in a rat excisional wound model and explored potential molecular mechanisms using a network-based bioinformatic approach. **Methods**: Twenty-one male Wistar rats were randomly assigned to three groups and treated twice daily for 14 days with a formulation containing 5% *Mentha spicata* essential oil diluted in olive oil, olive oil alone, or no treatment. Wound healing was assessed through macroscopic monitoring and histological scoring. The chemical composition of the essential oil was characterized using gas chromatography–mass spectrometry analysis. Predicted molecular targets of the major monoterpenes were analyzed through protein interaction networks and pathway enrichment analysis. **Results**: Macroscopic wound closure progressed in all groups by day 14. Histological analysis revealed that the olive oil group showed more advanced collagen deposition, re-epithelialization, and granulation tissue maturation, whereas the *Mentha spicata* group displayed a more pronounced inflammatory and proliferative histological pattern. Network-based analysis highlighted signaling pathways related to receptor-mediated cellular responses as potential molecular mechanisms associated with early inflammatory and proliferative processes. **Conclusions**: These findings suggest that the biological effects of *Mentha spicata* essential oil in wound repair may be phase-dependent and influenced by concentration and formulation. The results support further studies aimed at optimizing dose and delivery strategies for essential oil–based wound therapies.

## 1. Introduction

Wound healing is a highly regulated and dynamic biological process involving multiple cell types and molecular events that are coordinated across three overlapping yet distinct phases: inflammation, tissue regeneration, and tissue remodeling (maturation). The duration of these phases varies considerably, ranging from days to months or even years, depending on the content and severity of the injury. The primary goal of wound management is to promote the orderly progression of these phases and to prevent complications that may lead to chronic or non-healing wounds [1].

When a wound occurs, this layer may become infected and allow microorganisms, some of which live on the surface of the skin, to enter the bloodstream [1]. In response to tissue damage, dermal cells initiate collagen synthesis and support epithelial repair [2]. Dysregulated or prolonged inflammatory responses may delay wound closure and impair tissue remodeling. Proinflammatory cytokines, such as interleukin 1 beta (IL-1β), IL-6, tumour necrosis factor-alpha (TNF-α), and prostaglandin E2 (PGE2), are released by macrophages and are involved in the upregulation of inflammatory reactions, while wound healing is accelerated by appropriate temporal downregulation of proinflammatory cytokine levels [3]. Accordingly, the identification of therapeutic agents that can modulate inflammation while promoting tissue regeneration remains a major objective in wound healing research.

Essential oils are volatile, plant-derived mixtures of biologically active compounds that not only exhibit diverse therapeutic properties (including antimicrobial, antioxidant, and anti-inflammatory effects) but are also widely incorporated into cosmetic, fragrance, and food applications due to their functional and sensory roles [4]. Due to their complex chemical composition and bioactive effects on the skin, essential oils have been increasingly investigated in recent years for their potential roles in wound healing and skin repair [5]. *Mentha spicata* L., a member of the Lamiaceae family commonly known as spearmint, is a widely cultivated aromatic plant in Turkey and many other regions [6]. Phytochemical analyses documented in the literature indicate that carvone represents the principal constituent of *M. spicata* essential oil. It has been reported to contribute to wound healing through its antimicrobial properties and its capacity to modulate inflammatory responses. Moreover, carvone has been shown to exert anti-inflammatory activity by modulating inflammatory mediators, which may, in turn, support fibroblast-mediated tissue repair processes [7].

In addition to histological evaluation, in silico approaches have increasingly been employed to explore the potential molecular mechanisms underlying the biological effects of plant-derived compounds. Recent systems-level and gene-centered network analyses further support the utility of integrative bioinformatic approaches for elucidating complex, multi-target biological responses, particularly in inflammation and repair related pathways [8]. Network-based target prediction and pathway enrichment analyses allow the prioritization of signaling nodes and biological pathways that may be modulated by bioactive constituents, thereby providing a mechanistic framework that complements in vivo observations [9]. Given the complex chemical composition of *M. spicata* essential oil and the known pleiotropic activities of its major monoterpenes, we incorporated a network pharmacology-oriented bioinformatic analysis into the present study. Using in silico analyses followed by protein–protein interaction (PPI) network construction and pathway enrichment analysis, we aimed to identify putative molecular targets and signaling pathways potentially associated with the phase-specific wound healing responses observed histologically.

In the present study, we investigated the effects of topical *M. spicata* essential oil on cutaneous wound healing in a rat full-thickness excisional wound model, using olive oil alone and untreated wounds as comparators. The essential oil composition was characterized by GC–MS, and wound repair was assessed macroscopically and histologically (H&E and Masson’s trichrome) using a semi-quantitative scoring framework. To contextualize phase-oriented histological patterns, we additionally conducted a hypothesis-generating in silico target prediction and network-based analysis focused on the major monoterpenes identified by GC–MS.

## 2. Materials and Methods

### 2.1. Preparation of the Oil

The olive oil (fixed oil) was obtained from EGAŞ A.Ş., Ankara, Türkiye and the *M. spicata* essential oil was obtained from the company Orlife Global A.Ş. Doalinn, Bursa, Türkiye. The essential oil was diluted in fixed olive oil under appropriate conditions to obtain a final concentration of 5%. The concentration of *Mentha spicata* essential oil (5%) was selected based on previous experimental studies investigating the topical application of essential oils in wound healing models.

### 2.2. GC/MS-Based Chemical Analysis of the Essential Oil

Gas chromatography–mass spectrometry (GC–MS) analysis was performed using an Agilent Technologies 6890 gas chromatograph equipped with a 5973 N mass selective detector (MSD) (Agilent Technologies, Palo Alto, CA, USA). Chromatographic separation was achieved on an HP-5MS capillary column (30 m × 0.25 mm i.d., 0.25 μm film thickness; 5%-phenyl-methyl polysiloxane; Agilent J&W, Palo Alto, CA, USA). The oven temperature was initially held at 80 °C for 1 min and then increased at a rate of 20 °C/min to 240 °C, followed by a ramp of 5 °C/min to 260 °C and, finally, increased at 20 °C/min to 300 °C, where it was held isothermal for 10 min. The injector and detector temperatures were set at 250 °C and 300 °C, respectively. Helium was used as the carrier gas at a constant flow rate of 1 mL/min, with a split ratio of 1:10. The MSD operated in electron ionization (EI) mode at 70 eV, scanning a mass range of 30–300 amu. A 1 μL aliquot of the diluted essential oil sample (prepared by dissolving 10 μL of oil in 1 mL of dichloromethane, GC–MS only, not in vivo) was manually injected using an Agilent 7683B autosampler. Compound identification was conducted by comparing retention indices (RIs), calculated relative to a homologous series of n-alkanes (C9–C25), with values reported in the literature. Mass spectral data were matched against entries in the Wiley 9 (Wiley, New York, NY, USA) and NIST 17 (Gaithersburg, MD, USA) libraries. The relative percentage composition of each component was determined from the GC peak areas using the normalization method, without applying correction factors. The reported values represent the mean of three replicate GC–MS analyses. Relative peak area normalization was used to estimate composition; values represent relative abundance rather than absolute concentration due to compound-specific response factors. Accordingly, these values were used to define the major constituents for downstream hypothesis generation, not for absolute quantification.

### 2.3. Animals and Experimental Design

This study was conducted in the Experimental Research Laboratory of Ankara University Faculty of Medicine with the approval decision of Ankara University Animal Experiments Local Ethics Committee (decision number: 2025-09-92). Twenty-one male Wistar rats (Kobay A.Ş., Ankara, Turkey), aged 8 weeks and weighing between 250 and 300 g, were used. Animals were acclimatized to the laboratory environment for one week. They were housed under controlled environmental conditions with a 12 h light/dark cycle, stable temperature (21 ± 1 °C) and humidity (75 ± 5%), and ad libitum access to tap water and standard rat chow. Animals were monitored daily, and any notable changes in appearance or behavior were recorded. No dietary or water restrictions were applied. The rats were randomly assigned to three experimental groups (*n* = 7 per group). Group 1 was treated with common *M. spicata* essential oil diluted with olive oil, group 2 was treated with olive oil, and group 3, as a control group, was not treated with any application. Following general anesthesia induction (50 mg/kg ketamine HCl and 5 mg/kg Xylazine HCl, i.m.), the dorsal neck area of each animal was shaved and disinfected, and a 1.5 cm × 1.5 cm (2.25 cm^2^) square full-thickness excisional wound was created. The external appearance of the animals was regularly observed, and significant changes were recorded. No restrictions were applied to diet and water consumption. All the treatments were applied topically, covering all wound areas (~1 mL) twice daily for 14 consecutive days. The formulation was gently distributed across the wound surface and surrounding margins using a sterile gauze pad to ensure homogeneous coverage. The essential oil was diluted to a final concentration of 5% in olive oil as a carrier vehicle to improve topical tolerability. Throughout the experimental period, no signs of maceration, excessive moisture accumulation, or local irritation were observed at the wound site. No other drugs were used post-surgically. At the end of the treatment period, all animals were sacrificed, and samples were taken from the wound regions within surgical margins. The harvested tissues were separated for downstream analyses, with samples for histopathological evaluation fixed in 10% neutral-buffered formalin until processing.

### 2.4. Tissue Collection and Histological Analysis

At the end of the 14th day, the full-thickness excisional wound, along with the surrounding healthy skin tissue, was removed from all experimental groups. Skin samples were placed in 10% buffered formalin solution (pH: 7.3–7.4) for fixation. The fixed tissues were passed through a graduated alcohol series and transferred to xylene. The tissues were embedded in blocks incubated in paraffin in an oven at 56 °C. After routine procedures, 5 μm sections were taken from the blocks using a Leica RM2125RT (Leica Biosystems, Nussloch, Germany) microtome and prepared for staining. The preparations were stained with hematoxylin–eosin (HE) and Masson trichrome (MT) stains and examined under a light microscope (Zeiss Axio Scope A1, Carl Zeiss AG, Oberkochen, Germany). The tissue samples obtained from the experimental groups were evaluated based on six separate criteria, modified by us, using the Abramov histological scoring system and Scardno et al.’s histopathological research study, which are referenced in the literature [10]. This scoring method was previously developed by Zekavat et al. and is presented in Table 1. These criteria were categorized as inflammation, re-epithelialization, fibroblast proliferation, collagen deposition, neovascularization, and granulation tissue.

### 2.5. Statistical Analysis

All statistical analyses were performed by IBM SPSS for Windows, Version 22.0 (Armonk, NY, USA: IBM Corp). Multiple comparisons between groups were evaluated using one-way analysis of variance (Tukey’s post hoc), ANOVA. A *p*-value < 0.05 was considered statistically significant. The results were presented as mean ± standard error (SEM) for 7 rats in each group. The groups were compared for six histological parameters: inflammation, re-epithelialization, fibroblast proliferation, collagen deposition, neovascularization, and granulation tissue.

### 2.6. In Silico Target Prediction and Network Analysis

The in silico/network pharmacology workflow was restricted to the major GC–MS-identified constituents of the essential oil (carvone, limonene, and α-pinene) to generate tractable hypotheses from the dominant chemical fraction. For each compound (carvone, limonene, and α-pinene), predicted targets were retrieved using the 2019 version of SwissTargetPrediction (accessed on 12 January 2026), which is based on bioactivity data from ChEMBL version 23. The organism was set to Rattus norvegicus. Compound structures were provided as standard identifiers/representations (e.g., canonical SMILES); no geometry optimization, docking, or molecular dynamics simulations were performed in this study. The predicted target lists were exported, merged, and deduplicated to generate a unified candidate target set for downstream network analyses. To reduce selection bias, targets were not pre-filtered to wound healing gene sets; biological relevance was assessed post hoc based on enrichment and network topology (Supplementary Table S1). Protein–protein interaction (PPI) networks were constructed using STRING (https://string-db.org, accessed on 12 January 2026) based on the predicted targets of the major carvone, limonene, and α-pinene obtained via SwissTargetPrediction. Predicted targets were combined into a single candidate list for downstream network analysis.

To retain high-confidence interactions, STRING-derived interactions were filtered using a false discovery rate (FDR) cutoff of 1 × 10^−5^ and a minimum required interaction score of ≥0.4. The resulting interaction networks were visualized in Cytoscape (v3.10.3) [11]. Hub proteins were determined using the maximal clique centrality (MCC) algorithm implemented in the CytoHubba plugin (accessed on 12 January 2026). Reactome pathway enrichment analysis of the predicted target set (and/or the hub-centered module) was performed using the EnrichR web tool (https://maayanlab.cloud/Enrichr/, accessed on 12 January 2026), and the top enriched pathways were visualized according to −log_10_(*p*) value significance [12].

## 3. Results

### 3.1. Chemical Characterization of Essential Oil

The results of the chemical characterization of the essential oils are presented in Table 2. According to GC/MS analyses, *M. spicata* was confirmed to contain 79.06% carvone, 18.06% limonene, and 2.35% α-pinene as major constituents.

### 3.2. Photo Area Calculation Results

**Figure 1:**
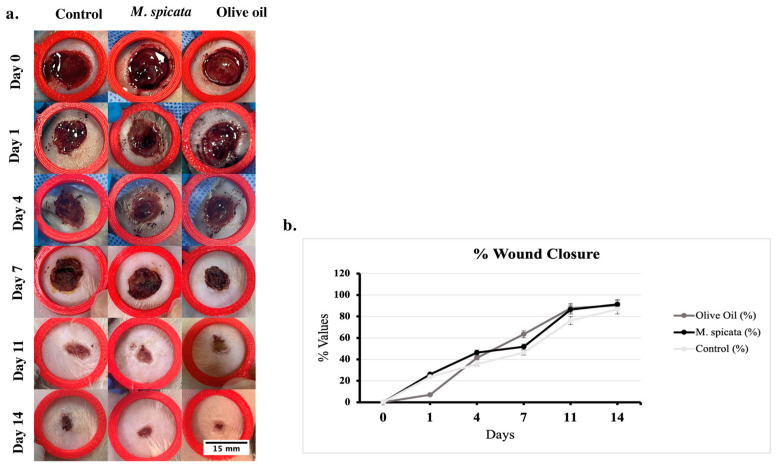
(**a**) Digital photographs of skin wound survival on postoperative days 0, 1, 4, 7, 11 and 14 in the control, olive oil, and *M. spicata* essential oil groups. (**b**) Wound closure areas in the wound tissue in the groups was calculated (in square meters) on days 0, 1, 4, 7, 11, and 14. Relative wound closure was shown as the percentage decrease in wound area compared to day 0. Wound closure rates were expressed as %.

Wound areas were recorded daily in all groups (control, olive oil-treated, and *M. spicata*-treated). Wound areas in photographs taken during the experiment were calculated with the ImageJ program (software version; 1.54g Wayne Rasband and contributors National Institutes of Health, USA) (Figure 1a). Wound closure area was calculated for all groups on days 0, 1, 4, 7, 11, and 14 (in square meters). Relative wound closure was calculated as the percentage reduction in the wound area compared to day 0. Wound closure rates were shown as %. At the end of day 14, the wound closure rate was calculated as 86.7% in the control group, 90.3% in the olive oil group, and 91.2% in the group treated with *M. spicata*. It was determined that the wound closure rates were similar at day 14, but the group treated with *M. spicata* had a higher wound closure area. Macroscopic evaluation of wound healing showed a progressive reduction in wound area in all groups over time. Both olive oil and *M. spicata*-treated wounds exhibited faster macroscopic closure compared to the control group, particularly at later time points (days 11 and 14). However, these observations were descriptive and intended to support histological findings rather than to provide quantitative statistical comparisons (Figure 1b).

### 3.3. Histopathological Evaluation

**Figure 2:**
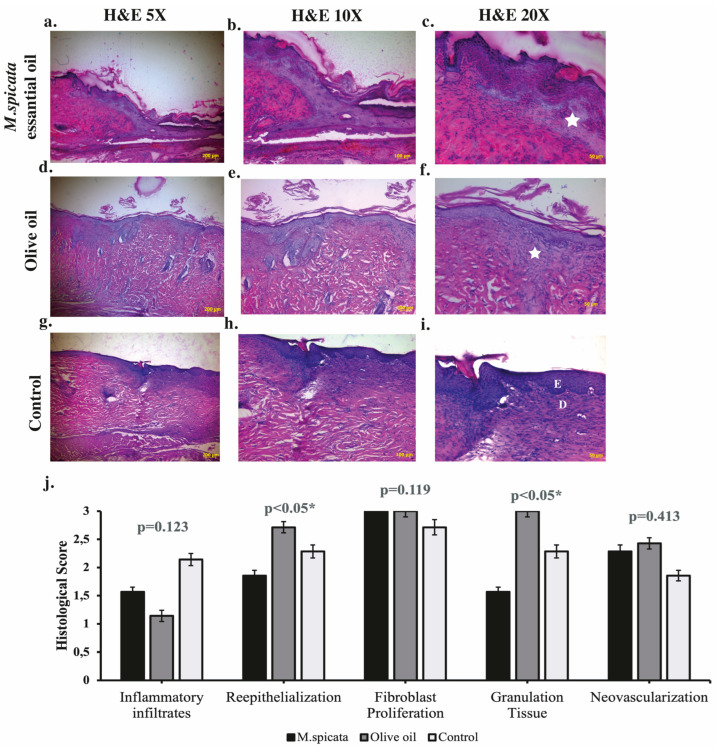
Histological analyses of wound healing. Light microscope images of wound tissue micrographs taken from animals that underwent punch biopsy. Representative hematoxylin and eosin (HE)-stained sections of wound areas from control, *M. spicata* oil-treated, olive oil, and control rats fourteen days after treatment. (**a**–**c**) *M. spicata* oil-treated group showing well-organized granulation tissue and re-epithelialization at increasing magnifications (5×, 10×, 20×; scale bars = 200 μm, 100 μm, 50 μm, respectively). (**d**–**f**) Olive oil group showing high granulation tissue. (**g**–**i**) Control group illustrating limited granulation and denser inflammatory infiltration. Asterisks indicate granulation tissue; D, dermis; E, epidermis. (**j**) Quantitative histological scoring comparing inflammatory infiltrates, re-epithelialization, fibroblast proliferation, granulation tissue amount, and neovascularization between control, olive oil-treated, and *M. spicata* essential oil-treated groups (* *p* < 0.05).

Microscopic evaluation of the wounds was performed using wound excision specimens that included the entire wound area along with its margins. Hematoxylin and eosin staining, a standard method in dermatopathology, was applied to assess key wound healing parameters such as inflammation, re-epithelialization, fibroblast proliferation, neovascularization, and granulation tissue (Figure 2). Comparative histopathological analysis between the groups was conducted on day 14 post-injury. Granulation tissue density score were also found to differ significantly between the groups (F = 5.769; *p* = 0.012). The olive oil group (3.0 ± 0.0) showed higher granulation tissue formation compared to the *M. spicata*-treated group (1.57 ± 0.97) (Figure 2f). This finding indicates that olive oil supports cellular regeneration and tissue integrity. Inflammatory cell infiltration score was not significantly between the groups (F = 2.362; *p* > 0.05). However, when observing the mean values, the higher inflammation level in the control group (2.14 ± 0.89) indicates the potential of *M. spicata* (1.57 ± 1.13) and olive oil (1.14 ± 0.38) to reduce the inflammatory response. The re-epithelialization score was significantly higher in the olive oil group (2.71 ± 0.49) compared to the *M. spicata*-treated group (1.85 ± 0.69) (F = 4.050; *p* = 0.035). This result demonstrates the supportive effect of olive oil on the regeneration of epithelial tissue. The neovascularization score showed an increasing trend in the olive oil group (2.42 ± 0.53) compared to the *M. spicata*-treated group (2.28 ± 0.75) and the control group (1.86 ± 1.07), although this difference was not statistically significant (F = 0.929; *p* > 0.05). Although no statistically significant difference was observed in fibroblast density among the groups (F = 2.400; *p* > 0.05), the higher mean values detected in both the *M. spicata* oil-treated group (3.00 ± 0.00) and olive oil group (3.00 ± 0.00) compared with the control group (2.78 ± 0.48) indicate a potential stimulatory effect of these oils on fibroblast activity. This trend suggests that *M. spicata* oil and olive oils may contribute positively to the tissue repair process by enhancing cellular components involved in wound healing.

**Figure 3:**
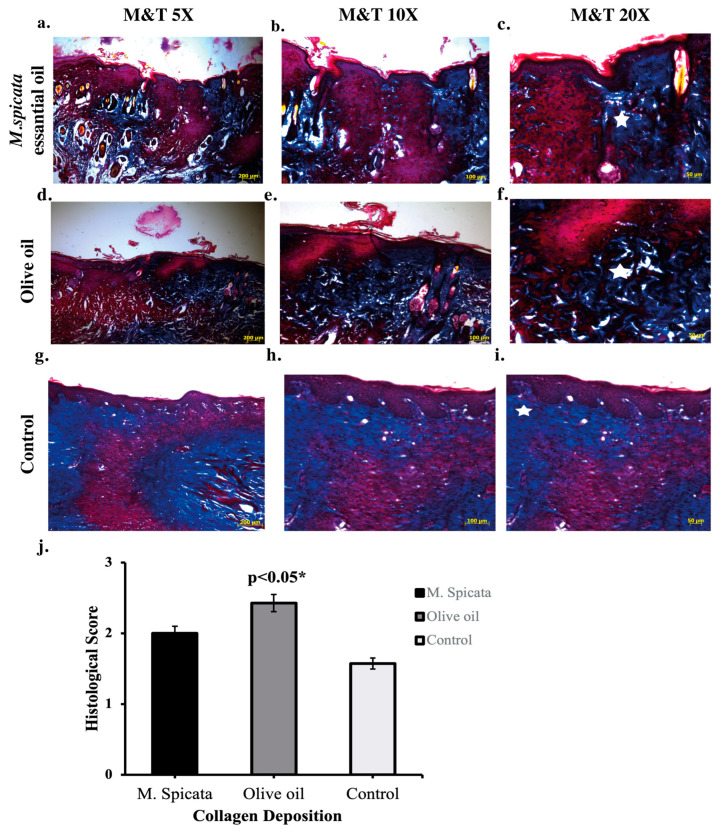
Histological analyses of collagen organization in wound healing. Light microscope images of wound tissue micrographs taken from animals that underwent punch biopsy. Representative Masson’s trichrome (MT)-stained sections of wounds from *M. spicata* oil-treated, olive oil-treated, and control rats fourteen days after treatment. (**a**–**c**) *M. spicata* oil-treated group showing high collagen deposition amount at increasing magnifications (5×, 10×, 20×; scale bars = 200 μm, 100 μm, 50 μm, respectively). (**d**–**f**) Olive oil group showing well-organized collagen deposition at increasing magnifications. (**g**–**i**) Control group illustrating limited collagen deposition. Asterisks indicate granulation tissue. In Masson’s trichrome staining, collagen fibers appear blue, muscle and cytoplasm are stained red, and nuclei are dark purple. (**j**) Quantitative histological scoring comparing collagen deposition between control, olive oil-treated, and *M. spicata* essential oil-treated groups (* *p* < 0.05).

Masson’s trichrome staining was used to assess collagen deposition remodeling, allowing for both morphological and semi-quantitative evaluation of tissue architecture during wound healing (Figure 3). A significant difference was found in the collagen accumulation parameter (F = 4.263; *p* = 0.031). Specifically, the higher collagen accumulation in the olive oil (2.42 ± 0.78) (Figure 3c) group and *M. spicata* essential oil group (2.00 ± 0.00) (Figure 3f) compared to the control group (1.57 ± 0.53) (Figure 3i) indicates that this oil has an effect that accelerates connective tissue formation in the wound area. Overall, the present findings indicate that the applied oils exerted differential effects on distinct phases of the wound healing process. Although the study was initially designed based on the hypothesis that *M. spicata* essential oil would demonstrate superior wound healing efficacy, the histopathological and statistical results revealed that olive oil produced a more prominent therapeutic effect, particularly in terms of re-epithelialization, collagen deposition, and maturation of granulation tissue. In contrast, *M. spicata* essential oil was associated with increased inflammatory infiltration and enhanced fibroblastic activity, suggesting a more pronounced influence on the inflammatory and proliferative phases rather than on tissue maturation and remodeling. Collectively, these results demonstrate that while olive oil predominantly supports the later stages of wound healing, *M. spicata* essential oil appears to modulate earlier biological events of the repair process.

Overall, day 14 histology suggested that olive oil primarily promoted late-stage maturation/remodeling (enhanced re-epithelialization, collagen deposition, and granulation tissue maturation), whereas the *M. spicata* essential oil formulation was associated with a relatively more inflammatory/proliferative tissue pattern. To explore potential signaling nodes that could plausibly relate to these phase-oriented histological phenotypes, we next performed an in silico target prediction and network-based analysis focused on the major essential oil monoterpenes identified by GC–MS.

### 3.4. In Silico Analysis of Target Prediction and Network Analysis

**Figure 4:**
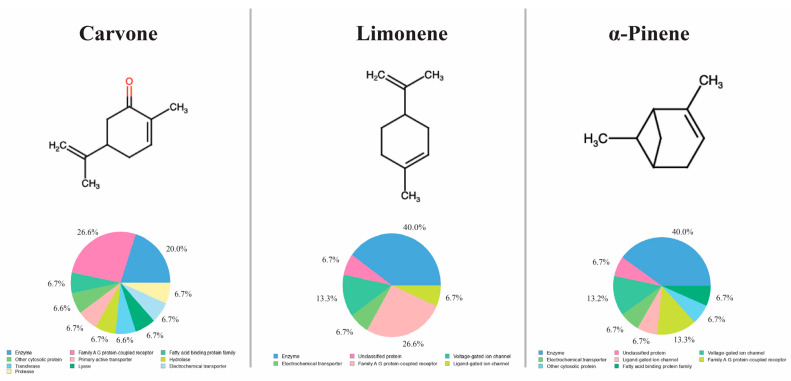
Chemical structures, predicted target class distribution, and network-based analysis of major *Mentha spicata* essential oil constituents. Chemical structures of carvone, limonene, and α-pinene are shown alongside the distribution of predicted target protein classes derived from SwissTargetPrediction analysis. Target prediction was performed for Rattus norvegicus. The workflow illustrates the integration of target prediction, STRING-based protein–protein interaction (PPI) network construction, Reactome pathway enrichment analysis, and hub protein identification using CytoHubba. Color coding for chemical structures follows standard CPK rules, where red indicates oxygen atoms.

SwissTargetPrediction analysis of carvone, limonene, and α-pinene identified a set of putative protein targets predominantly belonging to enzyme families, G protein-coupled receptors (GPCRs), and ligand-gated or voltage-gated ion channels. The distribution of predicted target classes differed among the three monoterpenes but consistently highlighted GPCR-related proteins as a major functional category (Figure 4).

The combined target list was subsequently used to generate a protein–protein interaction (PPI) network via STRING analysis, revealing a densely interconnected network indicative of functional relatedness among the predicted targets. Reactome pathway enrichment analysis of this network demonstrated significant overrepresentation of GPCR-associated signaling pathways, including Class A/1 (rhodopsin-like) receptors, GPCR ligand binding, signaling by GPCRs, amine ligand-binding receptors, and downstream Gα(i)-mediated signaling events. Additional enriched pathways were related to steroid metabolism and nuclear receptor-mediated transcriptional regulation.

**Figure 5:**
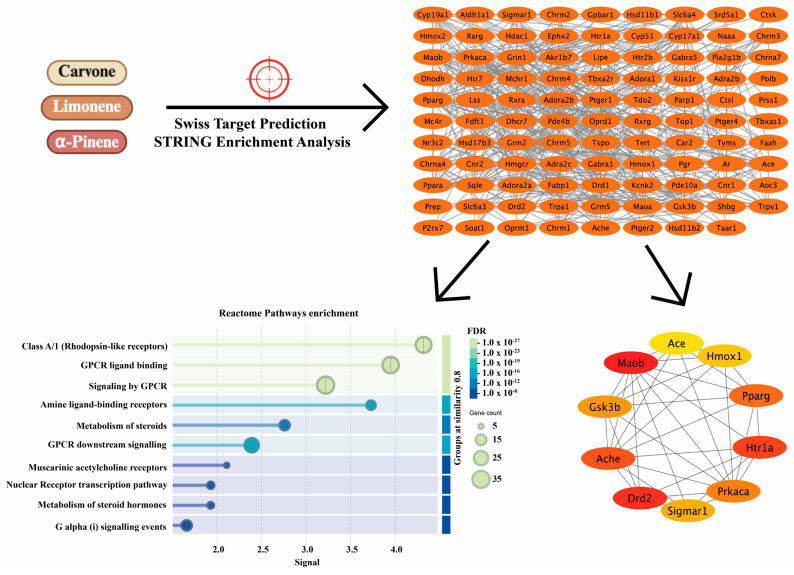
Protein–protein interaction network, Reactome pathway enrichment, and hub protein identification for predicted targets of *M. spicata* monoterpenes. The PPI network was constructed using STRING with a minimum interaction confidence score of ≥0.4. Reactome pathway enrichment analysis highlights GPCR-related signaling pathways, with dot size representing gene count and color indicating FDR-adjusted significance levels ranging from 1.0 × 10^−08^ to 1.0 × 10^−27^. The target symbols in the enrichment plot denote the precise signal position for each pathway. Hub proteins were identified using the maximal clique centrality (MCC) algorithm in CytoHubba, and the top-ranked hub nodes are visualized in the network diagram where the color gradient (red to yellow) indicates the ranking of nodes based on their MCC scores.

Hub analysis using the CytoHubba MCC algorithm identified several central nodes within the PPI network, including Drd2, Htr1a, Pparg, Prkaca, Sigma1, Ache, Maob, Gsk3b, Ace, and Hmox1. These proteins represent highly connected signaling nodes within the predicted interaction network and are functionally associated with neurotransmitter signaling, inflammatory modulation, metabolic regulation, and cellular stress responses (Figure 5).

## 4. Discussion

The present study was designed to demonstrate the superior wound healing efficacy of *M. spicata* essential oil; however, histopathological findings revealed that olive oil applied a more pronounced effect on re-epithelialization, collagen deposition, and granulation tissue maturation. In contrast, *M. spicata* oil was associated with increased inflammatory infiltration and fibroblastic activity, indicating a predominant influence on the inflammatory and proliferative phases rather than on tissue remodeling. These results underscore the importance of interpreting the biological effects of essential oils within the framework of composition- and concentration-dependent activity and suggest that different oils modulate distinct stages of the wound healing cascade.

The relatively higher variability observed in some histological parameters, particularly in inflammatory cell infiltration within the *Mentha spicata* group, may reflect heterogeneous biological responses among individual animals. Essential oils are complex mixtures of volatile bioactive compounds, and their local effects may vary depending on individual physiological sensitivity, wound microenvironment, and the dynamic nature of inflammatory processes during tissue repair. In addition, histological scoring systems represent semi-quantitative assessments, which may introduce a degree of variability when evaluating parameters such as inflammatory infiltration.

An increasing number of studies demonstrate that essential oils exert dose-dependent effects on wound healing, whereby low to moderate concentrations (typically 1–4%) promote anti-inflammatory activity, fibroblast proliferation, and re-epithelialization, whereas higher concentrations are more frequently associated with local irritation, edema, and excessive inflammatory cell infiltration. This pattern strongly supports the existence of a therapeutic window beyond which essential oils may shift from beneficial to irritant effects [11,12]. Consistent with this concept, preclinical studies employing relatively high concentrations, particularly in the range of 6–12%, have reported adverse local tissue reactions, including edema, exudation, and increased inflammatory infiltration, as confirmed by histological analyses [13]. In our study, *M. spicata* essential oil was formulated at a final concentration of 5% by dilution in fixed olive oil. When considered in the context of the concentration ranges reported in the literature, this dose lies at the upper boundary of the proposed therapeutic window and may partially explain the pronounced inflammatory response and enhanced fibroblastic activity observed in our histological findings. Therefore, our results further emphasize that the biological effects of essential oils on wound healing are not solely compound-specific but are also critically dependent on formulation and dosage, underscoring the importance of dose optimization in essential oil-based wound therapies.

In the present study, a single concentration (5%) of *Mentha spicata* essential oil was selected to ensure detectable biological activity while maintaining formulation stability in a topical preparation. Concentrations within the range of approximately 1–5% are frequently used in experimental studies investigating the topical application of essential oils in wound healing models. However, it is well recognized that the pharmacological effects of essential oils often exhibit non-linear dose–response relationships, where lower concentrations may exert anti-inflammatory or regenerative effects, whereas higher concentrations may induce local irritation or excessive inflammatory responses. Therefore, the inflammatory/proliferative histological pattern observed in the present study may partly reflect the use of a concentration positioned near the upper boundary of this commonly reported experimental range.

Beyond dose considerations, the dermatologic use of essential oils requires careful attention to safety, purity, and formulation quality, as topical preparations may elicit irritant or allergic contact dermatitis depending on the chemical profile and exposure conditions. Recent evidence syntheses in dermatology emphasize that essential oils can demonstrate meaningful bioactivity in inflammatory skin settings, yet their clinical utility is frequently constrained by tolerability issues, variability in composition, and differences in administered preparations [5]. In this study, our observation of increased inflammatory infiltration in the *M. spicata* group is compatible with the broader safety literature indicating that certain monoterpenes, particularly at higher local exposure, may shift tissue responses toward irritation rather than resolution.

Carvone was identified as the major component (72.8%) of *M. spicata* essential oil in a study by Biltekin et al. [14]. *M. spicata* from the Mediterranean region was shown to be rich in carvone (67.8%) and limonene (10.6%) in another study [15]. In samples of *M. spicata* from Pakistan, carvone (51.7%), cis-carveol (24.3%), and limonene (5.3%) were reported as predominant components, consistent with the seasonal variation described by Hussain et al. [16]. The GC–MS analysis of the *M. spicata* essential oil used in this study, which revealed a markedly high carvone content (79.06%), provides a reasonable mechanistic explanation for the observed histological response. At low to moderate concentrations, monoterpenes such as carvone are known to exert diverse biological activities; however, elevated local exposure has been associated with tissue irritation, sensitization, and enhanced recruitment of inflammatory cells [17]. The predominance of carvone, together with the relatively low abundance of potentially wound-supportive constituents such as linalool, may therefore have shifted the local tissue response toward irritation-mediated inflammation rather than orderly wound resolution [18]. The compositional imbalance in our study likely contributed to the persistence of proliferative activity and a delay in progression towards collagen maturation and tissue remodeling.

The phase-specific pattern observed in the *M. spicata* group is also consistent with experimental data on monoterpenes such as limonene, which may modulate early wound mediators and inflammatory dynamics. In murine models, topical D-limonene has been reported to influence key mediators involved in wound repair and to support aspects of tissue repair biology, although such effects can be context- and dose-dependent [19]. In our study, the relatively high proportion of carvone, together with the presence of limonene, may explain why *M. spicata* essential oil appeared to influence inflammatory and proliferative events (cell recruitment and fibroblastic activity) without leading to superior maturation outcomes, such as collagen organization and fully mature granulation tissue, by day 14.

In contrast, the olive oil group exhibited more organized collagen deposition, mature re-epithelialization with skin appendages, and reduced inflammatory infiltration. These findings align with reports demonstrating that phenolic compounds derived from olive oil possess anti-inflammatory and antioxidant properties, modulate fibroblast migration, and promote collagen maturation [20,21]. The favorable histological profile observed in this group supports the conclusion that the heightened inflammatory and proliferative response seen in the *M. spicata* essential oil-treated wounds was primarily attributable to the essential oil composition rather than to the vehicle or experimental protocol. The more favorable histological profile observed in the olive oil group is biologically plausible given the reported actions of olive oil phenolics on skin-relevant cell functions. Recent mechanistic work indicates that olive oil-derived phenolic compounds (e.g., hydroxytyrosol, tyrosol, and oleocanthal) can influence fibroblast behavior and wound-relevant processes, including migration- and extracellular matrix-associated responses [20]. Moreover, contemporary experimental evidence suggests that topical hydroxytyrosol can improve wound healing outcomes in metabolically compromised settings (e.g., diabetic models), supporting an anti-inflammatory/antioxidant rationale that aligns with our findings of reduced inflammatory infiltration and improved maturation parameters in the olive oil group [22]. These reports provide an external mechanistic framework for interpreting why olive oil outperformed *M. spicata* oil in parameters reflecting late-stage repair, such as collagen deposition and granulation tissue maturation.

The comparatively favorable outcomes observed in the olive oil group highlight the intrinsic biological activity of the vehicle itself, which has been reported to exert anti-inflammatory and antioxidant effects and to support fibroblast migration and extracellular matrix organization. In contrast, the histological pattern observed in the *Mentha spicata* group, characterized by relatively higher inflammatory and proliferative features, may suggest that the essential oil primarily modulates early wound healing dynamics rather than enhancing late-stage remodeling. This interpretation is consistent with the concept that certain phytochemical components may exert phase-specific effects during tissue repair, influencing early inflammatory responses without necessarily accelerating collagen maturation or granulation tissue organization at later stages.

Collectively, these results underscore the importance of optimizing essential oil concentration, compositional balance, and delivery strategy in wound healing applications [23]. Formulation approaches designed to regulate release kinetics and prevent abrupt high local monoterpene exposure may mitigate irritation and improve healing outcomes [24,25]. The present findings indicate that although *M. spicata* essential oil possesses intrinsic bioactivity relevant to wound repair, its application in a carvone-dominant formulation may prolong inflammatory and proliferative phases, thereby delaying complete tissue maturation.

Finally, formulation strategy is likely to be a critical determinant of whether *M. spicata* essential oil can be leveraged therapeutically without provoking irritation. The recent pharmaceutics-oriented literature highlights that incorporating essential oils into polymer-based scaffolds or controlled-release systems may improve local tolerability and sustain beneficial bioactivity by limiting abrupt high peak exposure at the wound site [26]. In parallel, recent topical formulation studies explicitly caution that increasing essential oil concentrations may compromise skin tolerability and recommend avoiding excessive doses due to irritation risk, an observation that resonates with the inflammatory profile observed here at a 5% formulation [27].

Our network-based bioinformatic analysis provides a mechanistic, hypothesis-generating framework to evaluate the phase-specific histopathological patterns observed in vivo. The enrichment of GPCR-associated pathways, amine ligand-binding receptors, and Gα(i)-mediated signaling suggests that the predicted molecular targets of carvone, limonene, and α-pinene are primarily embedded in receptor-mediated signaling networks that are critically involved in early inflammatory and proliferative stages of wound repair. Increasing evidence indicates that GPCR signaling orchestrates leukocyte aggregation, fibroblast migration, angiogenic responses, and cytokine release during the initial phases of tissue repair, thereby forming the quality and tempo of subsequent healing outcomes [28].

Consistent with this network profile, hub prioritization identified Drd2, Htr1a, Sigma1, Pparg, Prkaca, and Ache as central nodes within the predicted interaction network. Although these findings do not demonstrate direct molecular engagement in wound tissue, they support the growing concept that neurotransmitter-related and GPCR-coupled signaling pathways contribute to cutaneous repair by modulating inflammatory cell behavior, fibroblast activation, and cellular stress response programs. For instance, dopamine and serotonin receptor signaling has been shown to influence macrophage polarization, keratinocyte migration, and fibroblast proliferation, processes that are essential for effective wound closure yet require precise temporal regulation to prevent excessive inflammation or fibrosis [29,30]. Similarly, Sigma-1 receptor and PPAR-γ signaling are increasingly recognized as modulators of oxidative stress and inflammatory resolution, linking metabolic and neuroimmune pathways to tissue regeneration [31,32].

Importantly, this computational signature is broadly consistent with our day 14 histopathological pattern, in which the *M. spicata* group exhibited a more pronounced inflammatory and fibroblastic pattern without a parallel enhancement in remodeling-associated parameters such as collagen maturation and re-epithelialization; however, it remains hypothesis-generating in the absence of tissue-level molecular validation. This dissociation between early-phase cellular activation and late-stage tissue organization supports the interpretation that *M. spicata* essential oil may preferentially influence inflammatory proliferative signaling cascades rather than accelerating the remodeling phase of wound healing. Similar phase-dependent effects have been reported for other monoterpene-rich essential oils, where modulation of early inflammatory pathways does not necessarily translate into superior long-term structural outcomes [33,34].

Taken together, the in vivo findings and in silico predictions provide a plausible, hypothesis-generating context suggesting that the biological impact of *M. spicata* essential oil may be context- and phase-dependent. However, preferential engagement of specific pathways (e.g., GPCR-linked networks) remains speculative and requires independent tissue-level validation (e.g., RT-qPCR, Western blotting, or proteomics). These observations underscore the importance of integrating temporal endpoints and molecular readouts when evaluating phytotherapeutic agents for wound management.

While the present findings highlight the influence of *M. spicata* essential oil composition on wound healing outcomes, several limitations should be considered. First, only a single concentration (5%) of the essential oil was evaluated, which limits the ability to determine the optimal therapeutic window. Future studies investigating multiple concentrations would allow a more precise characterization of dose–response relationships and help identify the optimal balance between efficacy and local tolerability. Second, histological evaluation was performed only at day 14. Although this time point is widely used in experimental wound models to assess tissue maturation and collagen remodeling, earlier observations, such as at day 3 or day 7, would provide valuable insight into the inflammatory and proliferative phases of wound healing. Therefore, future studies including multiple time points would allow a more comprehensive understanding of the temporal effects of *Mentha spicata* essential oil during the wound healing process. Third, the in silico workflow focused on major monoterpenes and does not capture potential contributions of minor constituents or mixture-level synergistic/antagonistic interactions. Finally, because the computational approach is similarity-based rather than structure-based, it cannot resolve specific determinants of structure binding; such conclusions would require docking/molecular dynamics or experimental binding assays. Nevertheless, the current results provide a useful, hypothesis-generating framework to guide future formulation optimization and mechanistic validation.

## 5. Conclusions

This study investigated the effects of topical *Mentha spicata* essential oil on cutaneous wound healing in a rat excisional wound model and explored potential mechanisms using a network-based bioinformatic approach. Macroscopic analysis showed progressive wound contraction in all groups, with wound closure reaching approximately 85–90% by day 14. Histological evaluation demonstrated that the olive oil group exhibited more advanced collagen deposition, granulation tissue maturation, and re-epithelialization, whereas the *Mentha spicata* group displayed relatively higher inflammatory infiltration and fibroblastic activity.

These findings suggest that while olive oil may support later stages of tissue remodeling, *Mentha spicata* essential oil may preferentially influence the early inflammatory and proliferative phases of wound repair. Network-based analysis further indicated that major monoterpenes present in the essential oil may interact with signaling pathways involved in receptor-mediated cellular responses.

Overall, the results highlight the phase-dependent biological activity of *Mentha spicata* essential oil in wound healing and support further studies to optimize dose, formulation, and timing of topical application.

## Figures and Tables

**Table 1:** Histological parameters used to assess and calculate wound healing state.

Histological Parameters	Scoring System	Histological Grading
**Inflammatory infiltrates**	None	0
	Scant	1
	Moderate	2
	Abundant	3
**Re-epithelialization**	None	0
	Partial/marginal epithelium formation	1
	Complete but immature or thin (moderate)	2
	Complete and mature—with skin appendages (severe)	3
**Fibroblast proliferation**	Normal	0
	Mild increase	1
	Moderate increase	2
	Marked increase	3
**Collagen deposition**	None	0
	Scant, loose	1
	Moderate	2
	Abundant, organized	3
**Neovascularization**	None	0
	Up to 5 vessels per high-power field (HPF) (mild)	1
	6–10 vessels per high-power field (HPF) (moderate)	2
	>10 vessels per high- power field (HPF) (severe)	3
**Granulation tissue**	Immature	0
	Mild maturation	1
	Moderate maturation	2
	Fully matured	3

**Table 2:** Chemical composition of *Mentha spicata* essential oil.

Compound	Area	Relative Abundance (%)
Limonene	7,633,897	18.06
Menthol	55,133	0.13
Menthofuran	125,866	0.30
Linalool	42,229	0.10
a-Pinene	993,845	2.35
Carvone	33,407,686	79.06

## Data Availability

The original contributions presented in this study are included in the article. Further inquiries can be directed to the corresponding author.

## Supplementary Materials

The following supporting information can be downloaded at: https://www.mdpi.com/article/10.3390/biomedicines14040739/s1; Supplementary Table S1: GC-MS analysis result.

## Abbreviations

The following abbreviations are used in this manuscript:

MT	Multidisciplinary Digital Publishing Institute
HE	Hematoxylin and eosin
